# Adiposity, CVD risk factors and testosterone: Variation by partnering status and residence with children in US men

**DOI:** 10.1093/emph/eox005

**Published:** 2017-02-11

**Authors:** Lee T Gettler, Mallika S Sarma, Rieti G Gengo, Rahul C Oka, James J McKenna

**Affiliations:** 1 Department of Anthropology, University of Notre Dame, Notre Dame, IN, USA; 2 Eck Institute for Global Health, University of Notre Dame, Notre Dame, IN, USA; 3 Helen B. Kellogg Institute for International Studies, University of Notre Dame, Notre Dame, IN, USA

**Keywords:** marriage, father, men’s health, cardiovascular disease, androgens, NHANES

## Abstract

**Background and objectives:**

In many settings, partnered, invested fathers have lower testosterone than single men or fathers who are not involved in caregiving. Reduced testosterone has been identified as a risk factor for multiple chronic diseases, and men’s health also commonly varies by life history status. There have been few tests of whether variation in testosterone based on partnering and parenting has implications for men’s health.

**Methodology:**

We analysed data from a US population-representative sample (NHANES) of young-to-middle aged US men (*n* = 875; mean age: 29.8 years ± 6.0 [SD]). We tested for life history status differences in testosterone, adiposity levels and biomarkers of cardiovascular disease (CVD)-risk (HDL cholesterol; triglycerides; white blood cell count [WBC]).

**Results:**

Partnered men residing with children (RC) had lower testosterone and elevated abdominal adiposity compared to never married men not residing with children. While they did not significantly differ for WBC or triglycerides, partnered RC men also had comparatively lower HDL. Partnered RC males’ lower testosterone accounted for their relatively elevated adiposity, but testosterone, adiposity, and health-related covariates did not explain their relatively reduced HDL.

**Conclusions and implications:**

Our results linking life history status-based differences in testosterone and adiposity, alongside our complementary HDL findings, indicate that testosterone-related psychobiology might have implications for partnered RC men’s CVD risk in the US and other similar societal settings. These types of socially contextualized observations of men’s health and physiological function particularly merit incorporation in clinical discussions of fatherhood as a component of men’s health.

## BACKGROUND AND OBJECTIVES

Across vertebrates, testosterone (T) commonly plays a critical role as a physiological mechanism mediating male life history trade-offs through its facilitation of mating and competitive behaviors as well as its anabolic effects on energetically costly skeletal muscle tissue, its mobilization of stored energy reserves, and its modulatory interactions with the immune system [1–6]. Consistent with these roles for T in helping shape life history trade-offs, growing evidence supports the notion that human males’ T commonly declines when they engage in committed partnerships and invested fathering [7–10]. Studies indicate that reduced T in the context of these social relationships may help facilitate men’s engagement in sensitive, nurturing bonds and interactions and these predictions also generally apply to women [11, 12]. Low T may likewise diminish fathers’ tendencies towards reactive aggression and competitive-mating behaviors that could otherwise interfere with effective cooperative, nurturant partnering/parenting [3, 4, 11–16].

Scholars hypothesize that this psychobiological capacity emerged evolutionarily as invested fathers (alongside alloparents) cooperated with mothers to raise multiple, dependent young [3, 4, 6, 11–15, 17]. Bolstering this perspective, fathers have lower T during critical periods of male–female bonding and offspring development in multiple other vertebrate lineages in which biparental care has evolved, likely reflecting convergent evolutionary processes [3, 6, 11, 12, 15, 17, 18]. To date, there has been little consideration of the ways in which the psychobiology of partnering/parenting might help shape men’s health and how those effects may be dependent on the contextual expressions of those roles [8, 19, 20].

Compared to the more extensively studied behavioral and psychobiological corollaries of T in this domain, less research has evaluated its body composition and immune function implications based on partnering and parenting status [21, 22], which likely contribute to if or how life-history based variation in T affects men’s health across different ecological and societal contexts. Animal models, *in vitro* and experimental/clinical human studies, and observational research of subsistence-level societies with more marginal average nutrition and higher pathogen burdens, as would have been typical during human evolution, suggest that within-individual declines in T with partnering/parenting might benefit males by freeing up energy for enhanced investment in survival (energetic stores) and maintenance. Such reductions in T would also directly enable enhanced function of certain components of the immune system that are otherwise dampened by elevated T [2, 5, 23–26].

From a life history perspective, the notion that lower T is a plausible pathway enabling greater investment in survival and immune function is complementary to the idea that males have been selected to maintain higher T during their reproductive primes to prioritize potential fitness gains, at the expense of later-life longevity, which is likely reduced by prolonged, earlier life exposure to elevated T [24, 27–29]. While T’s interfaces with the immune system have been extensively reviewed elsewhere, various lines of evidence suggest that elevated T tends to suppress or dampen certain immune functions, such as aspects of the inflammatory response, B-cell development and differentiation, and costly forms of T cell-mediated immune responses [2, 21, 25, 26]. Meanwhile, the body also down-regulates T production during acute infection, reflecting a fundamental life history trade-off that adaptively prioritizes energetic investment in survival over reproduction [26].

In terms of T’s somatic effects, studies from diverse vertebrate taxa indicate that T helps anabolize energetically costly skeletal muscle tissue, playing a prominent role in sex-based and between-male differences in musculature and strength [5, 23]. Comparatively, there is somewhat mixed evidence for the applicability of this model to humans [22, 27]. Experimental and clinical research generally provides support for T’s anabolic effects on musculature [30]. Meanwhile, more naturalistic, observational studies show more modest support as well as null results, and a recent study of subsistence agriculturalists suggests that human strength and muscularity might be decoupled from T in the context of routine demanding physical exertion [22, 27]. When considering the notion that lower T promotes investment in energy stores, there are a number of well-documented bidirectional physiological pathways through which reduced T and elevated adiposity tend to coincide, within individuals [31–33]. For example, higher T prevents recruitment of adipose precursor cells, contributes to fat cells being utilized as energy substrates, and diverts metabolic resources towards building skeletal muscle, which itself then carries further basal energetic costs [33]. Meanwhile, in societies in which energetic abundance and sedentary lifestyles are common, men with greater adiposity often have reduced circulating T because adipose tissue converts (aromatizes) T to estradiol, and there is also evidence that obese men experience more rapid clearance of T from the body [31, 32].

In light of the adiposity-promoting and energy-sparing (through diminished anabolism of skeletal muscle) physiological effects of reduced T, we suggest there is a potential ‘mismatch’ between the past environments in which lower T among partnered, invested fathers first became common (evolutionarily) and the contexts in which that psychobiology finds expression today for some men in industrialized settings. This mismatch has potentially broad implications for men’s health; particularly, it may enhance their risk for cardiovascular disease (CVD). Under more evolutionarily relevant conditions of energetic constraint and higher pathogen burden, reduced T might be predicted to enhance longevity [24, 27–29]. Here, we hypothesize that reduced T might increase risk for certain chronic diseases (i.e. CVD) through its mechanistic effects on adiposity and inflammation for males in societies such as the US that are (on average) energy abundant, lower in pathogen loads, and more sedentary, reflecting evolutionarily anomalous conditions.

From an evolutionary perspective, our hominin ancestors were foragers, and it is likely that when men were invested in their partners and children, their (men’s) foraged and hunted calories would have benefitted their family (as well as others in the community) [34–38]. Among foraging societies in the ethnographic record, evidence shows that reproductive aged males are significant contributors to community energy budgets (often reflecting physically demanding and energetically costly labor) [34–38]. For example, studies of Hadza foragers in Tanzania show that when families have young, breastfeeding infants, fathers increase their time and effort in energetically costly subsistence [39] and unsurprisingly adults in this population are very physically active [40]. Meanwhile, Hadza fathers are commonly involved with caring for their children on a day-to-day basis and have lower T than non-fathers [41]. The latter observations are generally consistent with the overall cross-cultural patterns indicating that forager fathers spend the most time in close proximity to their children, compared to agriculturalists, pastoralists and horticulturalists [42]. Finally, forager diets are generally lean and their adiposity levels tend to be quite low, especially when compared to the average sedentary, overfed, non-pathogenically stressed resident of an industrialized population such as the US [38, 40, 43].

While there are limitations to insights on the evolutionary past that can be gleaned from modern hunter-gatherer data, we suggest that this cumulative perspective is consistent with the notion that if invested hominin (forager) fathers had reduced T [3, 14, 17], it might not have contributed to the accumulation of substantial adiposity, in light of other relevant lifestyle and energetic factors [40, 43]. Importantly, if lower T did enhance hominin fathers’ investments in adiposity (stored energy) it likely would have been beneficial by providing a buffer against energy short falls and increasing available metabolic resources to support maintenance of the immune system and its activation during active infection [23, 24, 40, 43–45]. In recent data from a community that practices intensive manual farming, Polish married men and fathers had elevated body fat compared to other men but T did not account for those body composition differences, despite T being lower among married men and fathers [22]. Elsewhere among nutritionally stressed Ariaal pastoralists in Kenya, men with lower adiposity had reduced T, likely reflecting the down-regulation of reproductive axes that can occur under conditions of extreme energetic constraint [23, 27, 46]. While the direction of the effects differs in these two studies, they are both consistent with notion that reduced T is not a primary contributor to adiposity in highly active populations, particularly if they face energetic stress.

In a slightly contrasting perspective, evidence from captive New World monkeys (common marmosets and cotton-top tamarins) in which fathers extensively care for their young has shown that expectant fathers add weight prior to their energetically costly twins and triplets being born. This weight gain helps buffer fathers against lost body mass due to the carrying costs of those litters and appears to be facilitated by endocrine changes (elevated prolactin) among fathers [15]. In total, there remain a number of unexplored, testable hypotheses related to the life history trade-off and health implications of T as it intersects with the variable demands of partnering/parenting in diverse ecological settings and under various subsistence practices.

For men in some industrialized populations, the transition to becoming an invested parent can also involve changes in physical activity, work hours, diet, and sleep, which we thus suggest might interrelate with declining T to negatively affect aspects of men’s health [47–50]. In contrast to demographic and epidemiological data, which often point to health-promoting effects of partnering (especially) and fathering (occasionally) [51–53], other studies have found that partnered men and fathers in ‘Western’ nations show increases in adiposity (e.g. body mass index [BMI], waist circumference) as well as biomarkers of cardiovascular (CVD) risk, compared to other men [49, 54–57]. These effects may occur through a number of behavioral pathways, including married men and fathers spending less time engaging in rigorous physical activity or more time in sedentary activity [49, 56, 57] and consuming less healthy diets, such as more overall dietary fat [47].

The health implications of between- and within-male T variation across the life course have likewise been particularly well studied in industrialized populations. Men with T in the lower range of normal or clinically low T are more likely to be overweight/obese, have elevated markers of inflammation, poorer profiles for cholesterol and triglycerides, as well as elevated risks for CVD and all-cause mortality [58–61]. Here, in light of the findings above relating partnering-parenting to reduced T as well as elevated CVD-risk factors, we focus primarily on these low-T related health implications.

In the present analyses, we analysed cross-sectional data from a large sample of young-to-middle aged adult men (*n* = 875; mean age: 29.8 years ± 6.0 [SD]) enrolled in the 2011–12 National Health and Nutrition Examination Survey (NHANES), which is a US-population representative sample. Stratifying men based on partnering and residence with children, which we use as a proxy for parenting status, we tested hypotheses regarding group differences in men’s adiposity levels and biomarkers of CVD risk (HDL cholesterol; triglycerides; white blood cell count [WBC]), which have also previously been linked to T [61, 62]. We specifically hypothesized that partnered men who were not residing with children and partnered men residing with children would have lower T, greater adiposity, lower HDL, elevated triglycerides and higher WBCs compared to never married men who were not residing with children. For outcomes that differed based on partnering and residence status, we then proceeded to test whether T was potentially in the pathway. Finally, we tested whether additional lifestyle and health-related factors represented mediating or confounding variables in our CVD-related analyses.

## METHODOLOGY

### Nhanes 2011–12

The US Centers for Disease Control and Prevention (CDC) conducts the NHANES data collections with the purpose of assessing health outcomes for a sample that is representative of the civilian, non-institutionalized US population. Here, we draw on data from the 2011–12 cross-sectional wave of the NHANES continuous collections. In total, we analysed data from 1602 reproductive aged US men between the ages of 20 and 60 years, but focus the majority of our analyses on the sub-sample of men (*n* = 875) between ages 20 and 40. See the online Supplementary Material file for our rationale for using 40 years of age as a cut-off point for these analyses as well as further details on the NHANES study design. 

### Total testosterone (T)

NHANES blood collection protocols involve single blood draws from subjects with the general timing (morning, afternoon, evening) recorded. Subjects’ blood samples were analysed for total T (ng/dl) at the National Center for Environmental Health (http://wwwn.cdc.gov/Nchs/Nhanes/2011-2012/TST_G.htm; last accessed 2/27/2017). We controlled for the time of sampling in all of our analyses predicting T. See online Supplementary Material file for further details.

### Biomarkers of CVD risk

Subjects’ total WBCs were analysed at the Mobile Examination Center (MEC) in which the blood draws took place. Men’s HDL-cholesterol (mg/dL) and triglycerides (mg/dL) were analyzed at the University of Minnesota Medical Center. Please see online Supplementary Material for further information. Detailed descriptions of the sampling criteria and laboratory procedures for all NHANES biomarkers can be found here: http://www.cdc.gov/nchs/nhanes/nhanes2011-2012/lab_methods_11_12.htm; last accessed 2/27/2017.

### Anthropometric variables

Participants’ waist circumference (cm), sagittal abdominal diameter (SAD; cm), height (m) and weight (kg) were measured using standard techniques. NHANES calculated subjects’ BMI from height and weight (kg/m^2^). We focus primarily on SAD in the present analyses (see online Supplementary Material). Further information on the anthropometric techniques used by NHANES can be found here: https://www.cdc.gov/nchs/data/nhanes/nhanes_11_12/Anthropometry_Procedures_Manual.pdf; last accessed 2/27/2017.

### Socio-demographic variables

For partnering status, we coded married and cohabitating men into a single category (‘partnered’) and similarly combined men who reported being divorced or separated into a single category, yielding a marital status variable with the following categories: married/cohabitating (‘partnered’), widowed, separated/divorced, never married. We did not include widowed individuals in these analyses (see online Supplementary Material).

In terms of ‘parenting’ status, men reported whether they were residing with children under the age of 18. We examined whether men were residing with children as a proxy for fatherhood status and created a variable that stratified men by both partnering and residence status (see online Supplementary Material). Men were also asked to identify whether they were specifically residing with young children (under 5 years of age) and/or older children (those between 6 and 18 years of age). We also controlled for a measure of socioeconomic status (education; see online Supplementary Material).

### Health-related behavioral variables

Participants reported their typical amounts of total sleep time, typical daily sedentary (seated) activity, and typical days and minutes (per bout) spent in moderate or vigorous recreational activity. We combined moderate and vigorous recreational activity into one variable (see online Supplementary Material). Men reported their average daily alcohol consumption over the past year. We categorized these data as heavy, moderate, or no drinking. Subjects also participated in dietary recalls in which they reported the food and beverages they consumed in the 24 h prior to the MEC interview. Using variables calculated by NHANES, we analysed subjects’ total caloric intake (kcal), dietary fat (g), and dietary sugar (g). Finally, men self-reported their general health on a five-point scale, which we combined into: poor/fair, good, and very good/excellent (see online Supplementary Material).

### Statistical analyses

Using survey design commands, we conducted all statistical analyses using Stata 14.0 (Stata Corporation). These commands prevent biased estimates and inaccurate significance levels by taking into account the complexities of the NHANES sample design (http://www.cdc.gov/nchs/tutorials/nhanes/SurveyDesign/SampleDesign/intro.htm; last accessed 2/27/2017). We also note that these survey design commands do not conduct correlation (e.g. Pearson’s *r*) analyses, thus we used regression techniques. For continuous dependent variables that approximated a normal distribution, we used OLS regression, while we used multinomial logistic regression for categorical outcomes and negative binomial regression for a right-skewed count variable (physical activity time).

We first conducted exploratory linear regression interaction analyses, testing whether men’s ages moderated the relationship between life history status (partnering and residence with children) and adiposity (SAD, waist circumference) and T, respectively. Then, drawing on men between 20 and 40 years of age, we assessed correlations (primarily using linear regression) between T and adiposity levels (SAD), respectively, in relationship to other anthropometric variables (waist circumference, BMI), CVD-related biomarkers (WBC, HDL, triglycerides), as well as health-related factors (sleep time, physical activity, sedentary activity, dietary intake, alcohol intake, and self-reported health); see Table 1. We next ran initial models testing whether T, adiposity levels, and CVD-related biomarkers differed based on life history status. We then evaluated associations between life history-related demographics (partnering, residence status, age of children) and those health-related factors (above). Thus, with these collective analyses for health-related factors, our goal was to identify independent variables that could have mediating, confounding or masking effects to those of our core analyses (on adiposity and CVD-related biomarkers) while also aiming to only include pertinent covariates and thus to avoid over-fitting the models.

**Table 1:** Assessing correlative relationships between biometric and health-related measures that have implications for CVD^a^

	Abdominal adiposity (SAD)			Total testosterone (T)		
Variables	β	95% CI	*P*-value	β	95% CI	*P*-value
**Anthropometrics**						
Waist circumference	**0.97**	**(0.94, 1.00)**	**0.0001**	**−0.46**	**(−0.55, −0.37)**	**0.0001**
BMI	**0.94**	**(0.89, 0.99)**	**0.0001**	**−0.44**	**(−0.53, −0.35)**	**0.0001**
SAD				**−0.46**	**(−0.53, −0.38)**	**0.0001**
**CVD-related biomarkers** ^a^						
HDL cholesterol	**−0.41**	**(−0.48, −0.35)**	**0.0001**	**0.25**	**(0.17, 0.32)**	**0.0001**
Triglycerides	**0.32**	**(0.20, 0.44)**	**0.0001**	**−0.28**	**(−0.39, −0.17)**	**0.0001**
White blood cell count	**0.27**	**(0.18, 0.36)**	**0.0001**	**−0.25**	**(−0.34, −0.15)**	**0.0001**
**Health-related covariates**						
Total sleep time	−0.05	(−0.13, 0.03)	0.208	−0.02	(−0.11, 0.07)	0.674
Weekly physical activity	**−0.12**	**(−0.16, −0.07)**	**0.0001**	**0.14**	**(0.03, 0.24)**	**0.013**
Weekly sedentary activity	0.02	(−0.06, 0.10)	0.565	−0.06	(−0.14, 0.03)	0.194
Total calories consumed	−0.01	(−0.13, 0.11)	0.853	0.04	(−0.06, 0.14)	0.406
Total dietary fat consumed	0.01	(−0.07, 0.10)	0.749	0.04	(−0.05, 0.14)	0.344
Total dietary sugar consumed	0.01	(−0.10, 0.12)	0.863	0.02	(−0.10, 0.13)	0.767
Current health (good)^b^	**−0.54**	**(−0.73, −0.35)**	**0.0001**	0.13	(−0.12, 0.37)	0.302
Current health (v. good/excellent)^b^	**−0.85**	**(−1.09, −0.61)**	**0.0001**	**0.31**	**(0.08, 0.53)**	**0.010**
Moderate alcohol consumption^c^	**−0.27**	**(−0.43, −0.11)**	**0.002**	−0.03	(−0.20, 0.13)	0.670
No alcohol consumption^c^	0.13	(−0.25, 0.52)	0.476	−0.09	(−0.36, 0.19)	0.503

a We converted all continuous variables to z scores. All models control for men’s ages and (for relevant biomarkers) timing of blood draw (not shown). Model results reflect SAD and T as (separate) dependent variables, with the exception of the ‘CVD-related biomarkers’, which we treated as dependent variables, predicted from SAD and T, respectively. Sample size: *n* = 875 unless noted below; waist circumference: *n* = 874; triglycerides: *n* = 416; dietary measures: *n* = 837; current health: *n* = 874; alcohol consumption: *n* = 818. Significant findings are listed in bold (all p < 0.05).

b Comparison group: men who reported being in poor/fair health.

c Comparison group: men who reported consuming heavy amounts of alcohol on a daily basis in the past year.

For SAD and CVD-related biomarkers that differed based on partnering and residence status in our initial models, we then proceeded to test whether T attenuated those associations. We also tested whether SAD (along with T) helped account for associations between partnering/residence status and CVD-related biomarkers. Finally, we added relevant health-related covariates to these models. We evaluated statistical significance at *P* ≤ 0.05. In the figures, we present values for the dependent variables, adjusted for covariates, using predictive margins following statistical models.

## RESULTS

**Figure 1.**
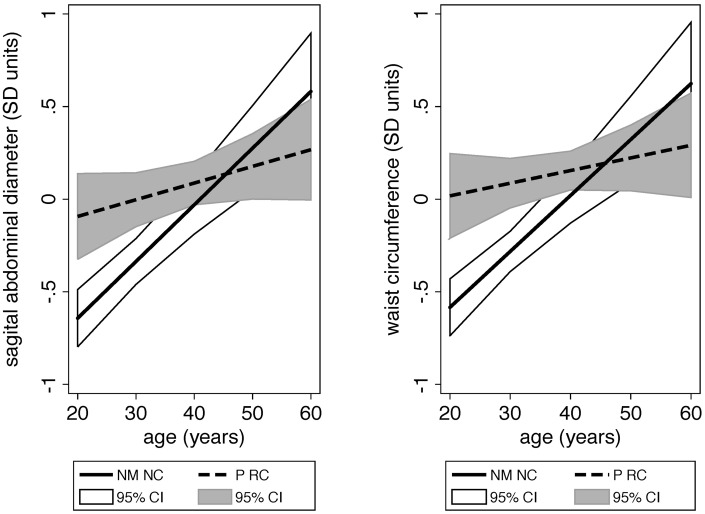
**(a, b)** Men’s predicted adiposity levels (SAD and waist circumference) based on their ages and stratified according to life history status These predicted adiposity outcomes result from moderation analyses (age × life history status in linear regression; see online Supplementary Table S2a), and we present the predicted values for never married men not residing with children (NM NC) and partnered men residing with children (P RC) for visual purposes. The interaction term for (partnered residence status × age) was highly significant (*P* = 0.001) for both SAD (*n* = 1602) and waist circumference (*n* = 1600). We report the full Results of the interaction models for all life history status categories in online Supplementary Table S2a. CI intervals represent 95% CIs

We first tested whether men’s ages moderated the relationship between life history status (LHS: partnering and residence with children) and T and adiposity (independently). As short hand we use the acronym ‘RC’ for men residing with children and ‘NC’ for men not residing with children. In the (LHS × age) interaction model predicting T, none of the interaction terms were statistically significant (all *P* > 0.5). Partnered RC had lower T than never married NC regardless of age (both *P* ≤ 0.01; online Supplementary Table S1a–c). For adiposity (waist circumference; SAD), the (LH × age) interaction terms for partnered NC and partnered RC were significant (all *P* < 0.05; Fig. 1; online Supplementary Table S2a). In age-separated analyses, younger partnered RC had greater adiposity, on average, relative to never married NC (both *P* < 0.01; online Supplementary Table S2b). However, among older men, never married NC had comparable adiposity to partnered men, regardless of their residence status (both *P* > 0.4; online Supplementary Table S2c). These patterns can be observed visually in Fig. 1. We focused our subsequent analyses on young-to-middle aged men (20–40 years of age).

We present descriptive statistics for that sub-sample of young-to-middle aged men in online Supplementary Table S3 and report the results of our initial correlational analyses in Table 1. Men with greater SAD had higher waist circumference and elevated BMI (*P* < 0.0001). Men’s adiposity (SAD, waist circumference and BMI) was also higher when their T was lower (all *P* < 0.0001; Table 1). Men who had greater adiposity (SAD) or lower T had reduced HDL cholesterol as well as elevated WBC and triglycerides (all *P* < 0.001; Table 1).

Testing for relationships between T, adiposity, and health-related covariates, we found that men who engaged in more frequent physical activity had higher T and lower adiposity (SAD), respectively (both *P* ≤ 0.01; Table 1). Men who reported their health as very good/excellent had higher T and lower adiposity than those in poor health (both *P* < 0.01), while subjects who drank in moderation had reduced adiposity compared to heavier drinkers (*P* = 0.002; Table 1). We conducted similar correlational analyses by LH status, but we only report results that overlap with these T/adiposity models, because of space limitations (see full results in online Supplementary Table S4a). Compared to never married NC, partnered NC engaged in less weekly physical activity (*P* = 0.007), but were also more likely to report being non-drinkers, versus more heavy consumers of alcohol (*P* = 0.018). Meanwhile, compared to men not residing with children, men living with older children were more likely to report that they were in poor/fair health compared to very good/excellent health and that they engaged in heavier drinking versus more moderate alcohol intake (both *P* ≤ 0.001; online Supplementary Table S4b).

**Figure 2.**
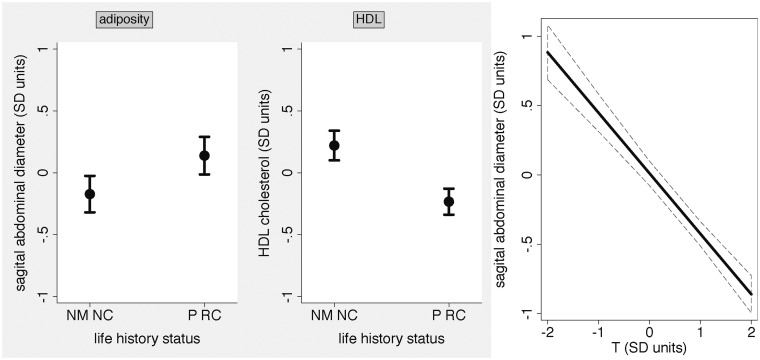
(**a**, **b**) Young-to-middle aged men’s adiposity levels SAD and HDL cholesterol levels predicted from their life history status. **(c)** Young-to-middle aged men s adiposity levels (SAD) predicted from testosterone (T) (a, b) We present the predicted values for never married men not residing with children (NM NC) and partnered men residing with children (P RC) for visual purposes. In the initial model, P RC men had higher SAD (*P* = 0.016) and lower HDL (*P* = 0.0001) than NM NC men (Tables 2 and 3). As shown in Models 1–3 in Tables 2 and 3, the results for SAD became non-significant (*P* = 0.248) after we added T to the model, whereas the results for HDL remained highly significant (*P* = 0.0003) with both T and SAD in the model. These linear regression analyses also controlled for age. Weighted means (±SD) for SAD (cm): NM NC men, 20.73 (±3.59); P RC men, 22.77 (±3.90). Weighted means (±SD) for HDL (mg/dl): NM NC men, 50.15 (±10.84); P RC men, 45.36 (±10.70). (c) Men’s SAD was lower when they had higher T (Tables 1–3; *P* ≤ 0.0001). We present the predicted values from a linear regression model that controlled for age and the timing of blood draw. Error bars and CI interval represent 95% CIs.

As we observed above, partnered RC had significantly lower T and greater adiposity than never married NC (both *P* < 0.01; online Supplementary Tables S1 and 2; Fig. 2a, b). Examining variation in CVD-related biomarkers, we found that partnered RC had lower HDL compared to never married NC (*P* = 0.0001; online Supplementary Table S5; Fig. 2), but the two groups did not differ for triglycerides or WBC (all *P* > 0.1; online Supplementary Table S5).

We then expanded from these models by testing whether relevant health-related variables and/or T attenuated relationships between men’s LH status and their adiposity and HDL profiles. With men’s self-reported health and physical activity time in the model, along with men’s ages and socioeconomic status (educational attainment), the effect size comparing SAD between partnered RC and never married NC diminished slightly (Table 2;*P* = 0.015). Adding T to the model (*P* < 0.0001; Fig. 2c), we found that partnered RC and never married NC no longer significantly differed for SAD (*P* = 0.248). Men’s physical activity time also no longer significantly predicted SAD (*P* = 0.296). Men’s current health status remained significant (*P* < 0.0001; Table 2), suggesting effects independent of T. Adding alcohol consumption to the model reduced the sample size but did not substantially affect the core results (not shown).

**Table 2:** Predicting men’s abdominal adiposity (SAD) from life history status, health-related variables and testosterone (*n* = 875)^a^

	Model 1			Model 2			Model 3			
Variables	β	95% CI	*P*-value	β	95% CI	*P*-value	β	95% CI	*P*-value	
**Life history status** **^b^**										
D NC	0.42	(−0.01, 0.85)	0.056	**0.43**	**(0.01, 0.86)**	**0.045**	**0.40**	**(0.05, 0.76)**		**0.028**
P NC	0.23	(−0.10, 0.56)	0.166	0.24	(−0.09, 0.57)	0.139	0.19	(−0.06, 0.45)		0.121
NM RC	0.07	(−0.18, 0.33)	0.560	0.02	(−0.24, 0.27)	0.872	−0.03	(−0.30, 0.24)		0.803
D RC	**0.33**	**(0.00, 0.66)**	**0.047**	0.11	(−0.14, 0.37)	0.358	0.08	(−0.16, 0.32)		0.499
P RC	**0.28**	**(0.06, 0.49)**	**0.016**	**0.27**	**(0.06, 0.49)**	**0.015**	0.14	(−0.11, 0.38)		0.248
**Health-related covariates**										
Weekly physical activity				**−0.08**	**(−0.11, −0.04)**	**0.0004**	−0.03	(−0.08, 0.03)		0.296
Current health (good)^c^				**−0.54**	**(−0.73, −0.35)**	**0.0001**	**−0.50**	**(−0.68, −0.31)**		**0.0001**
Current health (v. good/ excellent)^c^				**−0.82**	**(−1.07, −0.57)**	**0.0001**	**−0.69**	**(−0.90, −0.48)**		**0.0001**
**Testosterone**							**−0.41**	**(−0.48, −0.33)**		**0.0001**
*Model R2*		*0.082*			*0.147*			*0.304*	

a We converted all continuous variables to z scores, including the dependent variable (SAD). All models control for men’s ages and educational attainment, while model 3 also controls for timing of blood draw (not shown). Significant findings are listed in bold (all p < 0.05).

b Comparison group: men who were never married and not residing with children (*n* = 309).

c Comparison group: men who reported being in poor/fair health.

D NC, divorced not residing with children (*n* = 23); P NC, partnered not residing with children (*n* = 106); NM RC, never married residing with children (*n* = 77); D RC, divorced residing with children (*n* = 22); P RC, partnered residing with children (*n* = 338).

Focusing on men’s HDL, while controlling for men’s ages and education, we found that men who were more physically active and who reported good or very good/excellent health had elevated HDL (all *P* < 0.05; Table 3). Partnered RC had lower HDL than never married NC (*P* = 0.0001) in this model, although the effect size decreased slightly (Table 3). We then added T to the model. While men with elevated T had higher HDL (*P* = 0.0003), the findings for partnered RC again remained highly significant (*P* = 0.0004) but with a decrease in effect size (model not shown). After we included adiposity (SAD), which showed that men with greater adiposity had lower HDL (*P* < 0.0001), the effect size for partnered RC again declined somewhat but the result was highly significant (*P* = 0.0003; Table 3). With SAD included, T was no longer a significant predictor (*P* > 0.1; Table 3). Finally, the addition of alcohol consumption did not substantially alter the core findings (not shown).

**Table 3:** Predicting men’s HDL cholesterol from life history status, health-related variables, testosterone and adiposity (*n* = 875)^a^

	Model 1			Model 2			Model 3		
Variables	β	95% CI	*P-*value	β	95% CI	*P*-value	β	95% CI	*P*-value
**Life history status^b^**									
D NC	−0.48	(−0.99, 0.03)	0.065	−0.48	(−0.98, 0.02)	0.057	−0.32	(**−**0.71, 0.06)	0.096
P NC	**−0.32**	**(−0.64, −0.00)**	**0.050**	−0.29	(−0.61, 0.02)	0.065	−0.20	(**−**0.44, 0.04)	0.096
NM RC	−0.16	(−0.46, 0.15)	0.294	−0.14	(−0.45, 0.17)	0.352	−0.14	(**−**0.44, 0.17)	0.363
D RC	−0.42	(−0.95, 0.11)	0.111	−0.32	(−0.82, 0.19)	0.206	−0.28	(**−**0.76, 0.20)	0.236
P RC	**−0.45**	**(−0.64, −0.26)**	**0.0001**	**−0.44**	**(−0.63, −0.26)**	**0.0001**	**−0.33**	**(−0.48, −0.18)**	**0.0003**
**Health-related covariates**									
Weekly physical activity				**0.12**	**(0.06, 0.18)**	**0.001**	**0.08**	**(0.02, 0.15)**	**0.015**
Current health (good)^c^				**0.23**	**(0.01, 0.45)**	**0.039**	0.02	(**−**0.22, 0.25)	0.875
Current health (v. good/ excellent)^c^				**0.24**	**(0.05, 0.44)**	**0.018**	**−**0.08	(**−**0.27, 0.10)	0.337
**Testosterone**							0.05	(**−**0.02, 0.12)	0.127
**Abdominal adiposity (SAD)**							**−0.38**	**(−0.45, −0.30)**	**0.0001**
*Model R^2^*		*0.050*			*0.070*			*0.214*	

a We converted all continuous variables to z scores, including the dependent variable (HDL). All models control for men’s ages, educational attainment and timing of blood draw (not shown). Significant findings are listed in bold (all p ≤ 0.05).

b Comparison group: men who were never married and not residing with children (*n* = 309).

c Comparison group: men who reported being in poor/fair health.

D NC, divorced not residing with children (*n* = 23); P NC, partnered not residing with children (*n* = 106); NM RC, never married residing with children (*n* = 77); D RC, divorced residing with children (*n* = 22); P RC, partnered residing with children (*n* = 338).

## CONCLUSIONS AND IMPLICATIONS

Evolutionary and comparative-phylogenetic perspectives, particularly emerging from Wingfield and colleagues’ ‘Challenge Hypothesis,’ [63] have served as critical theoretical foundations to the fast-growing literature on the psychobiology of human partnering and parenting [3, 4, 11–13, 15, 16]. Given that invested human fathering and cooperative male–female partnerships are derived characteristics of the hominin lineage, a number of scholars have argued that men’s neuroendocrine capacity to flexibly down-regulate their T in those social contexts, particularly when they involve nurturance [11, 12], is likewise a related adaptive trait, although alternative explanations are possible [3, 4, 11–13, 15–17]. Here, we started from the premise that this psychobiological capacity has evolutionary roots and asked whether it is mismatched to contemporary expressions of partnering and residence with children for US men, particularly in light of their general lack of energetic stress and low pathogen burdens alongside potential health-related behavioral changes that might have combinatorial or additive effects with reduced T to increase adiposity and CVD risk.

Drawing on data from a large, nationally representative population of young-to-middle aged US men, we found evidence that is broadly consistent with that mismatch perspective. Specifically, partnered men residing with children (RC) had greater abdominal adiposity compared to never married men who were not residing with children (NC). We also found that the relationship between fatherhood and adiposity in RC men was attenuated after adjusting for T, consistent with T being in the pathway linking the two. To our knowledge, our findings are among the first to demonstrate that this well-documented pattern of lower T among partnered men and fathers (men residing with children, in our analyses) likely has direct biological implications for men’s health, particularly their CVD-risk.

Our study explicitly connects the various bodies of research on life history status, T, and adiposity [3, 4, 11–13, 15, 16, 49, 54–61] by showing that among young-to-middle aged US men, partnered RC males’ lower T strongly accounts for their higher abdominal adiposity, compared to never married NC men. The differences in measures of adiposity (waist circumference and SAD) between never married NC and partnered RC males were approximately 0.3 standard deviations (SD), suggesting a relatively robust relationship between the two. The effect sizes relating T to adiposity (across the entire sample) were also consistent with biologically meaningful effects. A 1-SD increase in T predicted a 0.4 SD reduction in adiposity (for each measure), which corresponds to 1.6 cm in SAD, 6.0 cm in waist circumference and 2.3 kg/m^2^ in BMI. These effect sizes point to potentially large health impacts, including for CVD risk (e.g. [64, 65]).

As we described previously, studies have documented multiple bidirectional physiological pathways through which lower T and elevated adiposity can cooccur within individuals [30–33]. Recent research has shown that US and European men tend to gain weight and adiposity when they become partnered or parents [49, 54–57], and longitudinal research on male psychobiology has demonstrated changes in men’s T across those transitions [7–10]. US men’s entry into fatherhood also often involves a suite of behavioral, dietary, and activity changes [47, 49, 56, 57], in addition to variation in T [9, 19, 66]. We think it is most likely that the tendency of partnered RC men in the US to have reduced T and elevated adiposity, on average, emerges through some combination of declining T through psychobiological pathways (and possibly other potential correlates of invested parenting, such as restricted or fragmented sleep [67]), decreased T-mediated mobilization of stored fats, and shifts in health-related behaviors, such as diet and physical activity, that can have additive effects on fat accumulation after these life history transitions. Because the NHANES data are cross-sectional, we cannot directly test this hypothesis regarding the timing of the effect, nor can we assess causality or directionality between men’s adiposity and their T.

In contrast to findings from relatively sedentary populations in high-income nations, there is less explicit evidence, to date, linking reduced T and elevated adiposity among males living under more energetically constrained ecological conditions [23, 27, 46, 68, 69]. This is likely largely or partially due to suppression of reproductive function when energy is sparse. Thus, when energy is limited males in better energetic condition tend to have higher T [23, 27, 46, 68]. The relevant studies that have examined these questions in relation to life history status or among fathers do not align with our results. They found no adiposity differences based on parenting status in spite of fathers’ lower T [70], no relationship between adiposity and fathers’ T production across the day [31], or elevated adiposity and lower T among fathers compared to non-fathers but not linkages between T and body fat [22]. Given that there are few studies in this area, it is unclear what accounts for this variation across contexts. An intriguing possibility is that differential relationships between T and adiposity across populations might partially reflect developmental programming effects related to early life experiences of energetic and/or pathogenic stress. This idea may merit further exploration in light of extensive research on early life programming and epigenetic regulation of metabolic functions [71, 72].

Returning to the present results, we found that partnered RC men also had lower HDL cholesterol than never married NC males. While adding T, adiposity, and health-related behaviors to the model diminished the strength of the relationship between life history status and HDL, it remained highly significant, with a biologically meaningful effect size. Although not directly comparable, our results for partnered RC men’s adiposity and HDL are most similar to a large clinical study of Italian men that found greater incidence of CVD events as men’s number of children increased, which was accounted for by elevated prevalence of metabolic syndrome [55]. In contrast, our results present a somewhat different picture from two prior studies showing a protective effect (against CVD mortality) of having 1–2 children, with increasing risks with more children [52, 53]. Those two studies focused on older men (most outside the window of producing children) and from a prior generation, compared to the sample we analysed. Those past results [52, 53] are complementary to our interaction results for adiposity, which showed that never married NC men have healthier body fat profiles but only for younger-to-middle aged men. In contrast, older (>40 years of age) never married NC subjects had elevated adiposity, particularly compared to their younger counterparts.

This could reflect changes in the effects of partnering and residence with children on adiposity via multiple health-related behavioral pathways as men age, perhaps protecting against fat accumulation among older men. From a life history trade-off perspective, an admittedly speculative possibility is that lower T during young adulthood and middle age, as a consequence of parenting and partnering, allows for enhanced concurrent investment in maintenance that then conveys health protective benefits that help mitigate adiposity accumulation later in life. Alternatively, it could reflect cohort effects, such as distinctive, variable roles associated with partnering and residing with children for the cohorts of older versus younger US men included in this cross-sectional study, leading to different implications for health. Thus, there are intriguing possibilities that merit exploration regarding how partnering and fatherhood relate to risk factors across the life course and potential cohort differences thereof due to cultural and economic shifts shaping familial roles [18].

To further explore the notion of a mismatch between T-related psychobiology and the demands and experiences associated with partnering and residing with children for US men, we tested whether health-related factors differed by life history-related demographics and whether they correlated with T or adiposity. These analyses yielded a limited set of results, in terms of their alignment with our hypotheses and the mismatch framework we proposed above. There were no differences between partnered RC and never married NC men for health-related factors that were also related to T or adiposity, although men living with older children reported being in poorer health and drinking alcohol more heavily than NC males. Partnered NC men were more likely to report not consuming alcohol and were also less physically active than never married NC males. We are hesitant to over-interpret the null results for these health-related analyses, which differ from some prior findings (e.g. see [14, 20, 51]). Among other factors, our non-significant findings could be due to the limitations of the non-prospective, cross-sectional data or methodological issues such as the imprecise and sparse measurement of sleep dynamics through self-reports as well as the lack of more specific data on the ages of coresidential children, i.e. residence with infants and toddlers (see below for further limitations).

When we included the relevant, significant health-related variables in the model predicting men’s adiposity (SAD), prior to including T, the effect size for the difference between never married NC and partnered RC men declined modestly. In a subsequent model, T explained the adiposity difference between never married NC and partnered RC males and also accounted for the relationship between physical activity and SAD. Of these health-related covariates, alcohol consumption (higher among men residing with older children) and men’s general health (poorer among men residing with older children) are potentially consistent with the mismatch model we have proposed here. The finding that some men residing with older children report poorer health, which is also linked with elevated adiposity, hints that other (unmeasured) factors associated with residence status (and likely fatherhood) accumulate to negatively impact men’s health. We also cannot rule out other possibilities, such as selection processes related to marriage and residence status or other confounding factors.

There are a number of limitations of our analyses that merit discussion. Because the NHANES continuous data collections are cross-sectional, we were not able to directly address within-individual change patterns in the present analyses, including between partnering and residence status, T, and CVD risk factors. While we cannot entirely rule out the possibility that, for example, men with reduced T and elevated adiposity are more likely to become partnered and reside with children, a number of longitudinal studies suggest that comparable life history transitions contribute to lower T and accumulation of adiposity, respectively, in at least some men [7–10, 54, 56]. Additionally, the T data for this study were measured from single blood samples from each subject, which raises concerns about statistical power and Type II errors. While repeated blood sampling from each subject would have increased the reliability of the T data, the precise laboratory procedure (mass spectrometry) and large sample sizes for our analyses help to allay these concerns.

In perhaps the most prominent limitation of the study, men reported whether they were currently residing with children, including separate questions regarding residence with young children (under 5 years of age) and older children (those between 6 and 18 years of age), but they were not asked any further information regarding the children’s ages or about their relationship with, relatedness to, or involvement with those children. Consequently, we cannot rule out that some of these men were potentially residing with children in non-parental contexts, such as living with their own younger siblings, grandchildren or other young relatives. While we think it is reasonable to propose that a majority of reproductive aged US men residing with children are likely serving in parental roles (biological-, adoptive-, foster-, step-parent), the familial demands and social dynamics of those different statuses for men’s T and health remain understudied and could likely vary [14, 73]. Finally, we could not model whether non-residential fatherhood had implications for the study’s outcomes. In spite of these limitations, we found a number of statistically significant associations between partnering and residence status, T and CVD risk factors with biologically meaningful effects sizes, which we think speak to the strength of their inter-relationships.

The total picture that emerges here is one in which young-to-middle aged US men who are partnered and residing with children have comparatively elevated adiposity that is linked to their lower T, which past research indicates could be explained by their nurturant, sensitive engagement in their families and that likely reflects psychobiological capacities with likely evolutionary origins [3, 4, 11–13, 15–17]. There is potential for the reduced T-elevated adiposity relationship to be exacerbated in the context of poor health and related behaviors (such as low levels of rigorous physical activity and heavy alcohol consumption), which are associated with greater body fat. Our results, considered alongside the complementary HDL findings, indicate that T-related psychobiology might play a contributing role in elevating US partnered fathers’ long-term risk for CVD, although longitudinal, prospective data that specifically focus on different fathering roles (e.g. biological-, step- and non-residential-fathering) are needed in this area. These types of socially contextualized observations of men’s health and reproductive physiological function particularly merit incorporation in clinical discussions of fatherhood and involvement with children as a component of men’s health [50].

## Supplementary Material

eox005_Supplementary_Data
