# Supplementary material for: Adiposity, CVD risk factors and testosterone: Variation by partnering status and residence with children in US men
Source: Evol Med Public Health. 2017 Feb 11;2017(1):67–80. doi: 10.1093/emph/eox005 (PMC5397396; doi:10.1093/emph/eox005)
Supplement: eox005_Supplementary_Data [file eox005_supplementary_data.docx]

**Supplemental Methods and Tables**

*NHANES 2011-2012*

The U.S. Centers for Disease Control and Prevention (CDC) conduct the NHANES data collections with the purpose of assessing health outcomes for a sample that is representative of the civilian, non-institutionalized U.S. population. The national population-representative characteristic of NHANES is made possible through a multi-stage, stratified, probability-cluster design. Here, we draw on data from the 2011-2012 cross-sectional wave of the NHANES continuous collections, during which 5000 men and women were interviewed in their homes and underwent a further medical examination. These exams include physiological and physical measurements as well as collection of blood samples and are conducted at mobile centers. Detailed information on NHANES sampling design and methodologies are publicly available through the CDC website: <http://www.cdc.gov/nchs/nhanes.htm>.

In total, we analyzed data from 1602 reproductive aged U.S. men between the ages of 20 and 60 years, which span the period from young adulthood to the age at which the overwhelming majority of men have ceased having biological children [1]. We originally anticipated exploring the framework we described in the Introduction, examining the interrelationships between T, CVD-related risk factors, and life history status, for all men between 20 and 60 years of age, as we did in a prior related analysis that focused on depression [2]. However, we also hypothesized that there might be age-based cohort differences in these cross-sectional NHANES data between younger and older men for the relationship between life history status and adiposity. Moreover, little is known about parenting status (residence with children in the NHANES data) and T for older men (but see [3]). Thus, we conducted initial analyses, to assess whether men’s ages moderated the relationship between life history status and adiposity and T (respectively). Based on these analyses, which we describe in the Results, we proceeded to test hypotheses focusing on *younger* men only (≤ 40 years of age; n = 875). Following the interaction models (age X life history status predicting adiposity; see Results), we chose 40 years of age as a cut point because U.S. men’s rate of fathering children declines dramatically from the third to fourth decade of life [1], thus this represents an age range (20-40 years) in which U.S. men’s children are most likely to be in their childhood and adolescent phases (i.e. highly dependent, in the U.S.) and perhaps maximally exacerbating aspects of the “mismatch” we propose in the Introduction and potentially positioning men for later life overweight/obesity and related morbidity, including CVD. Moreover, although scholarly definitions of “middle age” vary, this age range represents the period that aligns with men’s transition to middle-to-older age. Thus, processes-events in this period have implications for trajectories of later-life chronic disease. Finally, 40 years was also approximately the mean age for the broader sample (weighted mean: 39.97 years) and is approximately the upward age around which the moderation analyses suggest that never married men who do not reside with children still have lower adiposity than partnered men residing with children (see Figure 1a-b). We present descriptive statistics for the core analytical sample in Supplemental Table 3. Portions of our methodological descriptions in Supplemental Methods are adapted from Gettler and Oka’s [2] recent analyses of these 2011-2012 NHANES data.

*Total Testosterone (T)*

NHANES collects single blood samples from subjects and notes the general timing of their blood draws. Subjects’ blood samples were analyzed for total T (ng/dl) if they were six or more years old. Samples were measured for T at the National Center for Environmental Health using isotope dilution liquid chromatography tandem mass spectrometry according to the National Institute for Standards and Technology’s (NIST) reference method (http://wwwn.cdc.gov/Nchs/Nhanes/2011-2012/TST_G.htm). As reported on the NHANES website, “Stable Isotope-labeled testosterone is used as an internal standard to correct for sample recovery during the sample preparation process, [and] assay imprecision is 5%” (http://wwwn.cdc.gov/Nchs/Nhanes/2011-2012/TST_G.htm). Among subjects eligible for our analyses, the timing of their blood sampling was as follows: 50% morning, 31% afternoon, 19% evening, and we controlled for the time of sampling in all of our T-based analyses, because of T’s diurnal rhythm.

*Biomarkers of CVD-risk*

Subjects’ total white blood cell counts (WBC) were analyzed from their blood samples using a Coulter HMX Hematology Analyzer and were measured at the Mobile Examination Center (MEC) in which the blood draws took place. Men’s HDL-cholesterol (mg/dL) and triglycerides (mg/dL) were analyzed from blood samples using a Roche/Hitachi Modular P Chemistry Analyzer at the Collaborative Studies Clinical Laboratory at the University of Minnesota Medical Center. HDL was examined for adult participants whose blood was drawn at the MEC regardless of the time of day of the sampling, whereas triglycerides were only assessed for individuals who participated in morning MEC sessions and had fasted for 8.5 or more hours and less than 24 hours, hence the sample size for this analyte in our analyses (n = 416) is smaller (<http://wwwn.cdc.gov/nchs/nhanes/2011-2012/TRIGLY_G.htm>). Detailed information on the laboratory procedures for all NHANES biomarkers (including these CVD-related analytes and total T) can be found here: <http://www.cdc.gov/nchs/nhanes/nhanes2011-2012/lab_methods_11_12.htm>.

*Anthropometric variables*

Participants’ waist circumference (cm) and sagittal abdominal diameter (cm) (SAD) were measured using standard techniques. NHANES also calculated subjects’ body mass index (BMI) from height and weight (kg/m^2^). We focus primarily on SAD in the present analyses, as it is thought to provide a stronger anthropometric measure of abdominal visceral fat levels, which are particularly critical to health outcomes, such as CVD [4-5]. However, because SAD is a less commonly used anthropometric measure, we initially included both waist circumference and SAD in our moderation and correlation analyses, to show the findings extended to both variables (i.e. Table 1 and Supplemental Table 1). We also include BMI in the correlation analyses in Table 1 for reference. Further information on the anthropometric techniques used by NHANES can be found here: https://www.cdc.gov/nchs/data/nhanes/nhanes_11_12/Anthropometry_Procedures_Manual.pdf

*Socio-demographic variables*

For partnering status, we coded married and cohabitating men into a single category (“partnered”) and similarly combined men who reported being divorced or separated into a single category, yielding a marital status variable with the following categories: married/cohabitating (“partnered”), widowed, divorced/separated, never married. We did not include widowed individuals in these analyses because, among otherwise eligible subjects, there were few who were widowed (raw values, n = 2).

In terms of residence status, men reported whether they were residing with children under the age of 18. Men were not otherwise asked questions that could shed light on whether they were explicitly biological-, step-, or adoptive-fathers to those children or whether they had a non-parental relationship with them. Our aim was to use whether they were residing with children as a proxy for fatherhood status. However, to ensure that our phrasing more closely matches the NHANES phrasing, as short hand, we use the acronym “RC” for men residing with children and “NC” for men not residing with children. We analyzed the following demographic categories: never married not residing with children (“NC”), divorced NC, partnered NC, divorced residing with children (“RC”), never married RC, partnered RC. Men were asked to make a distinction between residing with young children (five years of age or under) and older children (those between six and 18 years of age). We created dichotomous variables indicating whether men were residing with young children (five years of age or under) or older children (older than five years). Finally, as a measure of socioeconomic status, we used the three-level NHANES education level variable: less than high school degree, high school graduate, more than high school.

*Health-related variables*

Participants reported their typical amounts of total sleep time, typical daily sedentary (seated) activity, and typical days (per week) and minutes (per day, when the activity occurs) spent in moderate or vigorous recreational activity. We combined moderate and vigorous recreational activity into one variable (typical days of moderate/vigorous activity X minutes per day, when such activity occurs). Men reported their average daily alcohol consumption over the past year. The CDC defines “heaving drinking” as having 15+ drinks per week. Thus, we created a categorical variable with the following categories: heavy drinking (3+ drinks per day), moderate drinking (2 or fewer drinks per day), or no drinking (zero drinks per day). Finally, men self-reported their general health on a five-point scale, which we combined into: poor/fair, good, and very good/excellent.

*References*

[1] Martin JA, Hamilton BE, Osterman MJK, Curtin SC, Matthews TJ. Births: final data for 2013*. National Vital Statistics Reports* 2015; **64:**1-68.

[2] Gettler LT ,Oka RC. Are testosterone levels and depression risk linked based on partnering and parenting? Evidence from a large population-representative study of US men and women*. Soc Sci Med* 2016; **epub:**.

[3] Pollet TV, Cobey KD , van der Meij L. Testosterone levels are negatively associated with childlessness in males, but positively related to offspring count in fathers*. PLoS ONE* 2013; **8:**e60018.

[4] Tchernof A ,Despres JP. Pathophysiology of human visceral obesity: an update*. Physiol Rev* 2013; **93:**359-404.

[5] Öhrvall M, Berglund L , Vessby B. Sagittal abdominal diameter compared with other anthropometric measurements in relation to cardiovascular risk*. Int J Obes* 2000; **24:**497-501.

| **Supplementary Table S1a. Predicting men’s testosterone (T) from life history status × age (n = 1602)^a^** | | | |
| --- | --- | --- | --- |
|  | **β** | **95% CI** | **p value** |
| **main effects** |  |  |  |
| D NC | -0.25 | (-1.17, 0.66) | 0.565 |
| P NC | -0.26 | (-0.91, 0.38) | 0.400 |
| NM RC | -0.11 | (-0.91, 0.68) | 0.765 |
| D NC | -0.23 | (-1.29, 0.84) | 0.659 |
| P RC | -0.64 | (-1.20, -0.07) | 0.029 |
| age | -0.25 | (-1.17, 0.66) | 0.565 |
| **interaction terms** |  |  |  |
| D NC **×** age | 0.004 | (-0.01, 0.02) | 0.690 |
| P NC **×** age | 0.003 | (-0.01, 0.02) | 0.655 |
| NM RC **×** age | -0.001 | (-0.03, 0.03) | 0.942 |
| D RC **×** age | 0.002 | (-0.02, 0.03) | 0.904 |
| P RC **×** age | 0.008 | (-0.01, 0.02) | 0.235 |
| *model R2* | *0.080* | | |
| ^a^we converted T to z scores. All models control for timing of blood draw (not shown). Comparison group: men who were never married and not residing with children (NM NC). D: divorced/separated. P: partnered. NM: never married. NC: not residing with children. RC: residing with children. | | | |

| **Supplementary Table S1b. Predicting men’s testosterone (T) from life history status for men 20 to 40 years old (n = 875)^a^** | | | |
| --- | --- | --- | --- |
|  | **β** | **95% CI** | **p value** |
| D NC | -0.03 | (-0.45, 0.39) | 0.890 |
| P NC | -0.13 | (-0.45, 0.20) | 0.430 |
| NM RC | -0.14 | (-0.38, 0.10) | 0.230 |
| D NC | -0.17 | (-0.47, 0.13) | 0.254 |
| P RC^b^ | **-0.34** | **(-0.55, -0.14)** | **0.003** |
| *model R2* | *0.073* | | |
| ^a^we converted T to z scores. All models control for men’s ages and timing of blood draw (not shown). Comparison group: men who were never married and not residing with children (NM NC). D: divorced/separated. P: partnered. NM: never married. NC: not residing with children. RC: residing with children.  ^b^Weighted means (± SD) for T (ng/dl): NM NC men, 463.76 (± 168.56); P RC men, 394.02 (± 149.16). | | | |

| **Supplementary Table S1c. Predicting men’s testosterone (T) from life history status for men 41 to 60 years old (n = 727)^a^** | | | |
| --- | --- | --- | --- |
|  | **β** | **95% CI** | **p value** |
| D NC | -0.14 | (-0.50, 0.22) | 0.410 |
| P NC | -0.16 | (-0.47, 0.15) | 0.291 |
| NM RC | -0.15 | (-0.65, 0.35) | 0.533 |
| D NC | -0.11 | (-0.50, 0.27) | 0.542 |
| P RC^b^ | **-0.21** | **(-0.37, -0.05)** | **0.013** |
| *model R2* | *0.070* | | |
| ^a^we converted T to z scores. All models control for men’s ages and timing of blood draw (not shown). Comparison group: men who were never married and not residing with children (NM NC). D: divorced/separated. P: partnered. NM: never married. NC: not residing with children. RC: residing with children.  ^b^Weighted means (± SD) for T (ng/dl): NM NC men, 422.32 (± 134.80); P RC men, 377.65 (± 156.79). | | | |

| **Supplementary Table S2a. Predicting men’s adiposity (SAD and waist circumference) from life history status × age^a^** | | | | | | | |
| --- | --- | --- | --- | --- | --- | --- | --- |
|  | **abdominal adiposity (SAD)**  **(n = 1602)** | | |  | **waist circumference**  **(n = 1600)** | | |
|  | **β** | **95% CI** | **p value** |  | **β** | **95% CI** | **p value** |
| **main effects** |  |  |  |  |  |  |  |
| D NC | 0.26 | (-0.77, 1.29) | 0.599 |  | 0.25 | (-0.58, 1.08) | 0.535 |
| P NC | **0.64** | **(0.12, 1.16)** | **0.018** |  | **0.60** | **(0.07, 1.14)** | **0.030** |
| NM RC | -0.17 | (-0.90, 0.55) | 0.623 |  | -0.12 | (-0.89, 0.64) | 0.740 |
| D NC | 0.29 | (-0.76, 1.33) | 0.570 |  | 0.56 | (-0.46, 1.59) | 0.264 |
| P RC | **0.98** | **(0.49, 1.48)** | **0.001** |  | **1.07** | **(0.52, 1.62)** | **0.001** |
| age | **0.03** | **(0.02, 0.04)** | **0.0001** |  | **0.03** | **(0.02, 0.04)** | **0.0001** |
| **interaction terms** |  |  |  |  |  |  |  |
| D NC **×** age | 0.002 | (-0.02, 0.02) | 0.763 |  | 0.00 | (-0.02, 0.02) | 0.912 |
| P NC **×** age | **-0.01** | **(-0.03, -0.00)** | **0.019** |  | **-0.01** | **(-0.02, -0.00)** | **0.042** |
| NM RC **×** age | 0.01 | (-0.02, 0.03) | 0.448 |  | 0.01 | (-0.02, 0.03) | 0.667 |
| D RC **×** age | **-0.004** | **(-0.03, 0.02)** | **0.755** |  | **-0.01** | **(-0.04, 0.01)** | **0.352** |
| P RC **×** age | **-0.02** | **(-0.04, -0.01)** | **0.005** |  | **-0.02** | **(-0.04, -0.01)** | **0.006** |
| *model R2* | *0.127* | | |  | *0.117* | | |
| ^a^we converted SAD and waist circumference to z scores. Comparison group: men who were never married and not residing with children (NM NC). D: divorced/separated. P: partnered. NM: never married. NC: not residing with children. RC: residing with children. | | | | | | | |

| **Supplementary Table S2b. Predicting men’s abdominal adiposity (SAD) from life history status for men 20 to 40 years old (n = 875)^a^** | | | |
| --- | --- | --- | --- |
|  | **β** | **95% CI** | **p value** |
| D NC | **0.51** | **(0.03, 0.98)** | **0.037** |
| P NC | 0.21 | (-0.12, 0.53) | 0.193 |
| NM RC | 0.13 | (-0.08, 0.34) | 0.198 |
| D NC | **0.43** | **(0.08, 0.77)** | **0.018** |
| P RC | **0.31** | **(0.11, 0.51)** | **0.004** |
| *model R2* | *0.070* | | |
| ^a^we converted SAD to z scores. All models control for men’s ages (not shown). Comparison group: men who were never married and not residing with children (NM NC). D: divorced/separated. P: partnered. NM: never married. NC: not residing with children. RC: residing with children. | | | |

| **Supplementary Table S2c. Predicting men’s abdominal adiposity (SAD) from life history status for men 41 to 60 years old (n = 727)^a^** | | | |
| --- | --- | --- | --- |
|  | **β** | **95% CI** | **p value** |
| D NC | **0.43** | **(0.03, 0.84)** | **0.036** |
| P NC | -0.02 | (-0.30, 0.25) | 0.857 |
| NM RC | -0.18 | (-0.59, 0.23) | 0.368 |
| D NC | -0.08 | (-0.45, 0.28) | 0.633 |
| P RC | -0.09 | (-0.36, 0.18) | 0.487 |
| *model R2* | *0.041* | | |
| ^a^we converted SAD to z scores. All models control for men’s ages (not shown). Comparison group: men who were never married and not residing with children (NM NC). D: divorced/separated. P: partnered. NM: never married. NC: not residing with children. RC: residing with children. | | | |

| **Supplementary Table S3. Sample characteristics (n = 875)^a^** | | |
| --- | --- | --- |
|  | **Mean** | **SD** |
| **demographic characteristics** |  |  |
| age (years) | 29.84 | 5.96 |
| education |  |  |
| % < high school graduate | 16.4 | - |
| % high school graduates | 52.7 | - |
| % > high school graduate | 30.9 | - |
| **marital status** |  |  |
| % married/cohabitating | 52.8 | - |
| % divorced/separated | 5.1 | - |
| % never married | 42.1 | - |
| **residence with child** |  |  |
| % living with child | 46.1 | - |
| % living with young child (≤ 5 years old) | 30.3 | - |
| % living with older child (>5 years old) | 31.3 | - |
| **biomarker values** |  |  |
| total testosterone (ng/dl) | 432.45 | 165.46 |
| HDL cholesterol (mg/dl) | 47.46 | 11.14 |
| white blood cell count (1000 cells/ul) | 6.86 | 1.97 |
| triglycerides^b^ (mg/dl) | 122.58 | 73.58 |
| **anthropometrics** |  |  |
| waist circumference (cm)^b^ | 96.35 | 15.02 |
| sagittal abdominal diameter (cm) | 21.85 | 3.96 |
| body mass index (kg/m^2^) | 27.76 | 5.67 |
| **general health^b^** |  |  |
| % poor/fair | 9.3 | - |
| % good | 35.3 | - |
| % very good/excellent | 55.4 | - |
| **health-related behaviors** |  |  |
| total sleep time (hours) | 6.79 | 1.19 |
| weekly sedentary activity (min) | 364.98 | 193.97 |
| weekly rigorous physical activity (min) | 250.10 | 335.15 |
| alcohol consumption^b,c^ |  |  |
| heavy alcohol consumption | 50.6 | - |
| moderate alcohol consumption | 36.8 | - |
| no alcohol consumption | 12.6 | - |
| total calories consumed (kcal)^b^ | 2785.93 | 1105.62 |
| total dietary fat consumed (gm)^b^ | 103.10 | 49.40 |
| total dietary sugar consumed (gm)^b^ | 146.52 | 89.93 |
| ^a^Descriptive statistics analyzed using Stata’s survey design commands (see Methods).  ^b^sample sizes: triglycerides, n = 416; waist circumference, n = 874; general health, n = 874; alcohol consumption, n = 818; dietary measures, n = 837).  ^c^see definitions for these categories in the Supplemental Methods. | | |

| **Supplementary Table S4a. Assessing correlative relationships between life history status and health-related measures that have implications for CVD^a^** | | | | | | |
| --- | --- | --- | --- | --- | --- | --- |
|  | **partnered NC^a,b^** | | | **partnered RC^a,b^** | | |
|  | **β** | **95% CI** | **p value** | **β** | **95% CI** | **p value** |
| **health-related covariates** |  |  |  |  |  |  |
| total sleep time | 0.03 | (-0.34, 0.39) | 0.881 | -0.12 | (-0.31, 0.07) | 0.196 |
| weekly sedentary activity | -0.13 | (-0.43, 0.16) | 0.356 | **-0.51** | **(-0.76, -0.25)** | **0.001** |
| total calories consumed | 0.04 | (-0.35, 0.43) | 0.830 | 0.13 | (-0.10, 0.36) | 0.250 |
| total dietary fat consumed | 0.17 | (-0.27, 0.61) | 0.437 | **0.24** | **(0.09, 0.38)** | **0.003** |
| total dietary sugar consumed | 0.27 | (-0.42, 0.97) | 0.420 | 0.23 | (-0.08, 0.53) | 0.133 |
|  | **IRR** | **95% CI** | **p value** | **IRR** | **95% CI** | **p value** |
| weekly physical activity | **0.75** | **(0.62, 0.92)** | **0.007** | 0.85 | (0.66, 1.11) | 0.213 |
|  | **RRR** | **95% CI** | **p value** | **RRR** | **95% CI** | **p value** |
| current health (good)^c^ | 1.31 | (0.41, 4.19) | 0.634 | 0.98 | (0.39, 2.45) | 0.964 |
| current health (v. good/ excellent)^c^ | 1.56 | (0.57-4.29) | 0.368 | 0.76 | (0.29, 2.01) | 0.562 |
| moderate alcohol consumption^d^ | 1.61 | (0.79, 3.28) | 0.177 | 1.41 | (0.80, 2.48) | 0.215 |
| no alcohol consumption^c^ | **2.57** | **(1.20, 5.53)** | **0.018** | 1.65 | (0.71, 3.81) | 0.226 |
| ^a^we converted all continuous variables to z scores, with the exception of weekly physical activity. All models control for men’s ages. NC: not residing with children. RC: residing with children.  ^b^The comparison group for all analyses in this table is men who were never married and not residing with children. Results for other life history status categories not shown. Model results reflect the “health-related covariates” as dependent variables and life history status as an independent variable. IRR: incidence rate ratio from negative binomial regression models. RRR: relative risk ratios from multinomial logistic regression models. Sample size: n = 875 unless noted below.  dietary measures: n = 837; current health: n = 874; alcohol consumption: n = 818.  ^c^comparison group: men who reported being in poor/fair health.  ^d^comparison group: men who reported consuming heavy amounts of alcohol on a daily basis in the past year. | | | | | | |

| **Supplementary Table S4b. Assessing correlative relationships between ages of fathers’ children and health-related measures that have implications for CVD^a^** | | | | | | |
| --- | --- | --- | --- | --- | --- | --- |
|  | **fathers of young children^a,b^** | | | **fathers of older children^a,b^** | | |
|  | **β** | **95% CI** | **p value** | **β** | **95% CI** | **p value** |
| **health-related covariates** |  |  |  |  |  |  |
| total sleep time | -0.19 | (-0.38, 0.01) | 0.058 | 0.01 | (-0.17, 0.19) | 0.923 |
| weekly sedentary activity | -0.15 | (-0.38, 0.07) | 0.162 | **-0.39** | **(-0.56, -0.23)** | **0.0001** |
| total calories consumed | **0.17** | **(0.06, 0.28)** | **0.005** | 0.02 | (-0.14, 0.19) | 0.760 |
| total dietary fat consumed | **0.19** | **(0.06, 0.31)** | **0.007** | 0.03 | (-0.11, 0.18) | 0.623 |
| total dietary sugar consumed | **0.19** | **(0.02, 0.36)** | **0.034** | 0.10 | (-0.09, 0.28) | 0.281 |
|  | **IRR** | **95% CI** | **p value** | **IRR** | **95% CI** | **p value** |
| weekly physical activity | 0.87 | (0.63, 1.19) | 0.347 | 1.06 | (0.87, 1.29) | 0.560 |
|  | **RRR** | **95% CI** | **p value** | **RRR** | **95% CI** | **p value** |
| current health (good)^c^ | 0.66 | (0.36, 1.20) | 0.160 | 0.83 | (0.46, 1.48) | 0.498 |
| current health (v. good/ excellent)^c^ | 0.86 | (0.48, 1.51) | 0.571 | **0.30** | **(0.16, 0.53)** | **0.0001** |
| moderate alcohol consumption^d^ | 1.37 | (0.79, 2.36) | 0.243 | **0.53** | **(0.37, 0.75)** | **0.001** |
| no alcohol consumption^c^ | 1.07 | (0.43, 2.66) | 0.868 | 1.08 | (0.63, 1.83) | 0.775 |
| ^a^we converted all continuous variables to z scores, with the exception of weekly physical activity. All models control for men’s ages.  ^b^The comparison group for all analyses in this table is men who were not residing with children. Model results reflect the “health-related covariates” as dependent variables and life history status as an independent variable. IRR: incidence rate ratio from negative binomial regression models. RRR: relative risk ratios from multinomial logistic regression models. Sample size: n = 875 unless noted below.  dietary measures: n = 837; current health: n = 874; alcohol consumption: n = 818.  ^c^comparison group: men who reported being in poor/fair health.  ^d^comparison group: men who reported consuming heavy amounts of alcohol on a daily basis in the past year. | | | | | | |

| **Supplementary Table S5. Predicting men’s HDL cholesterol, triglycerides, and white blood cell counts (WBC) from life history status^a^** | | | | | | | | | |
| --- | --- | --- | --- | --- | --- | --- | --- | --- | --- |
|  | **HDL**  **(n = 875)** | | | **triglycerides**  **(n = 416)** | | | **WBC**  **(n = 875)** | | |
|  | **β** | **95% CI** | **p value** | **β** | **95% CI** | **p**  **value** | **β** | **95% CI** | **p value** |
| **life history status^b^** |  |  |  |  |  |  |  | | |
| D NC | -0.49 | (-0.99, 0.01) | 0.056 | -0.19 | (-0.67, 0.29) | 0.409 | 0.26 | (-0.14, 0.66) | 0.184 |
| P NC | **-0.32** | **(-0.64, -0.00)** | **0.050** | 0.10 | (-0.16, 0.36) | 0.435 | -0.19 | (-0.41, 0.03) | 0.090 |
| NM RC | -0.19 | (-0.49, 0.11) | 0.198 | -0.15 | (-0.35, 0.05) | 0.127 | 0.05 | (-0.16, 0.26) | 0.614 |
| D RC | -0.43 | (-0.97, 0.10) | 0.106 | 0.34 | (-1.00, 1.68) | 0.602 | -0.21 | (-0.67, 0.24) | 0.332 |
| P RC | **-0.47** | **(-0.66, -0.28)** | **0.0001** | 0.14 | (-0.15, 0.44) | 0.317 | 0.08 | (-0.10, 0.27) | 0.351 |
| *model R2* |  | *0.049* |  |  | *0.036* |  | *0.070* | | |
| ^a^we converted all continuous variables to z scores. All models control for men’s ages and the HDL and WBC models control for timing of blood draw (not shown).  ^b^comparison group: men who were never married and not residing with children. D: divorced/separated. P: partnered. NM: never married. NC: not residing with children. RC: residing with children. | | | | | | | | | |
